# 

**Published:** 1956-04

**Authors:** 


### THE MEDICAL JOURNAL OF THE SOUTH-WEST

*Incorporating*
*The Bristol Medico-Chirurgical Journal*

ARMED FORCES
MAY - 7 1956
MEDICAL LIBRARY

*April 1956*

VOL. 71 (ii). No. 260 Price 4/-

